# Feasibility, acceptability and potential effectiveness of an information technology-based, pharmacist-led intervention to prevent an increase in anticholinergic and sedative load among older community-dwelling individuals

**DOI:** 10.1177/2042098618805881

**Published:** 2018-10-30

**Authors:** Helene G. van der Meer, Hans Wouters, Martina Teichert, Fabiënne Griens, Jugoslav Pavlovic, Lisa G. Pont, Katja Taxis

**Affiliations:** University of Groningen, Unit PharmacoTherapy, -Epidemiology & -Economy, HPC: XB45, Antonius Deusinglaan 1, 9711 AV Groningen, the Netherlands; University Medical Centre Groningen, Groningen, The Netherlands; Leiden University Medical Centre, Leiden, The NetherlandsRoyal Dutch Pharmacists Association, The Hague, The Netherlands; Foundation for Pharmaceutical Statistics, The Hague, The Netherlands; University of Groningen, Groningen, The Netherlands; University of Technology Sydney, Sydney, New South Wales, Australia; University of Groningen, Groningen, The Netherlands

**Keywords:** aged, deprescribing, drug burden index, hypnotics and sedatives, medical informatics, medication review, muscarinic antagonists, pharmacists

## Abstract

**Background:**

Anticholinergic/sedative medications are frequently used by older people, despite their negative impacts on cognitive and physical function. We explore the feasibility, acceptability and potential effectiveness of an innovative information technology (IT)-based intervention to prevent an increase in anticholinergic/sedative load in older people.

**Methods:**

This was a prospective study in 51 Dutch community pharmacies. Pharmacists used an IT-based tool to identify patients aged ⩾65 years, with existing high anticholinergic/sedative loads (drug burden index ⩾2) and a newly initiated anticholinergic/sedative medication. We determined the following. Feasibility: number of eligible patients identified. Acceptability: pharmacists’ satisfaction with the intervention, pharmacists’ time investment and patients’ willingness to reduce medication use. Potential effectiveness: number of recommendations, rate of agreement of general practitioners (GPs) with proposed recommendations and factors associated with agreement. To evaluate the latter, pharmacists conducted medication reviews and proposed recommendations to GPs for 5–10 patients selected by the IT-based tool.

**Results:**

We included 305 patients from 47 pharmacies. Feasibility: a mean of 17.0 (standard deviation, 8.8) patients were identified per pharmacy. Acceptability: 43 pharmacists (91.5%) were satisfied with the intervention. The median time investment per patient was 33 min (range 6.5–210). Of 35 patients, 30 (85.7%) were willing to reduce medication use. Potential effectiveness: pharmacists proposed 351 recommendations for 212 patients (69.5%). GPs agreed with recommendations for 108 patients (35.4%). Agreement to stop a medication was reached in 19.8% of recommendations for newly initiated medications (37 of 187) and for 15.2% of recommendations for existing medications (25 of 164). Agreement was more likely for recommendations on codeine [odds ratio (OR) 3.30; 95% confidence interval (CI) 1.14–9.57] or medications initiated by a specialist (OR 2.85; 95% CI 1.19–6.84) and less likely for pharmacies with lower level of collaboration with GPs (OR 0.15; 95% CI 0.02–0.97).

**Conclusion:**

This innovative IT-based intervention was feasible, acceptable and potentially effective. In one-third of patients an increase in anticholinergic/sedative load was prevented within reasonable time investment.

## Background

Medications with anticholinergic or sedative properties are of great concern in older people. They have a negative impact on cognitive and physical function and increase the risk of falls, dementia, hospitalization, and mortality.^1–3^ Despite these risks, anticholinergic and sedative medications are frequently prescribed to older people.^4,5^ Interventions to reduce the anticholinergic/sedative load among older individuals are urgently needed. One strategy that has been proposed for reducing this load is a pharmacist-led medication review. This is ‘a structured, critical examination of a patient’s medicines with the objective of reaching an agreement with the person about treatment, optimizing the impact of medicines, minimizing the number of medication related problems and reducing waste’.^6^ A few studies evaluated the effect of a pharmacist-led medication review on chronically used anticholinergic/sedative medications. While two small Australian studies found positive effects,^7,8^ we found pharmacist-led medication reviews to have no effect on deprescribing chronically used anticholinergic/sedative medications in a recent randomized controlled trial across 15 Dutch community pharmacies.^9^

Information technology (IT)-based interventions targeting newly initiated medications are another approach that potentially may reduce anticholinergic/sedative load. Since deprescribing chronically used anticholinergic/sedative medications is difficult, using IT to identify patients with newly initiated medications and performing a medication review to prevent an increase in anticholinergic/sedative load may be more successful. The use of IT-based approaches to identify patients with potentially ineffective or harmful medication use is increasing.^10^ In Dutch community pharmacy practice, pharmacists already use IT-based drug therapy alerts to monitor the safety of medication use in electronic patient records (e.g. detecting drug–drug interactions, contraindications, dosing in patients with renal impairment).^11^ Thus, using IT to identify older individuals with newly initiated anticholinergic/sedative medication is worthwhile to explore.

We built an innovative IT-based pharmacist-led intervention to prevent an increase in anticholinergic/sedative load among older Dutch community-dwelling individuals. In line with best practice for the development and evaluation of such a complex healthcare intervention,^12^ in this study we tested the feasibility, acceptability and potential effectiveness of this IT-based pharmacist-led intervention.

## Methods

### Study design and setting

The study was conducted in 51 community pharmacies located throughout the Netherlands in both rural and urban areas between September and December 2017. At each pharmacy, one pharmacist participated in the study. All participating pharmacists were enrolled in the national 2-year post-graduate programme to become a pharmacist specialized in community pharmacy. Participation in this study was part of their specialization training. Pharmaceutical care is well established in the Netherlands. This includes patient counselling for newly initiated medications, drug–drug interaction monitoring, and performing medication reviews. Pharmacies operate a pharmacy information system with a complete electronic medication history of their patients, as each individual patient is registered with a single pharmacy.^13^ Furthermore, Dutch pharmacists routinely collaborate with the general practitioners (GPs) in the area. This includes routine contact (phone or face-to-face meetings) to discuss individual patients and regular pharmacotherapy audit meetings.^14^ The Medical Ethical Committee of the University Medical Centre of Groningen confirmed that the study did not fall under the scope of the Medical Research Involving Human Subjects Act.

### IT-based tool to identify eligible patients

Each pharmacist ran an online report module containing an algorithm based on the patient inclusion criteria (described below) in their pharmacy information system to obtain a list of eligible patients. The module was developed by the Dutch Foundation for Pharmaceutical Statistics (SFK). The SFK has access to anonymous pharmacy dispensing data from the pharmacy information system of more than 95% of the Dutch community pharmacies; they collect these data to analyze national drug utilization and to provide pharmaceutical services.^15^

Eligible patients were aged ⩾65 years and received a newly prescribed potentially inappropriate anticholinergic/sedative medication in the past month. A newly prescribed medication was defined as a medication, or a medication with a similar action (World Health Organisation Anatomical Therapeutical Chemical (ATC) code level 3 or 4)^16^ dispensed for the first time in a 12-month period. We screened for those newly prescribed anticholinergic/sedative medications that were known to be potentially inappropriate in older people, including benzodiazepines, bladder antimuscarinics, tricyclic antidepressants, opioids, classic antihistamines, antipsychotics, second-generation antidepressants and a few cardiovascular medications. For these medications evidence-based guidance on prescribing in older people was available.^17,18^ Furthermore, patients needed to have a total cumulative anticholinergic/sedative load above a predefined threshold value of 2, according to the drug burden index (DBI). The DBI is a measure of total cumulative anticholinergic/sedative load and was calculated as $\mathrm{DBI}=\sum^{\;}\frac{D}{D+\delta }$ where, D = daily dose and δ = the minimum recommended daily dose.^19^ The recommended daily dose was determined according to Dutch Pharmacotherapeutic reference sources.^20,21^ All medications with potential anticholinergic/sedative properties were included in the calculation. As there is no consensus internationally regarding which medications are considered to have anticholinergic properties,^22^ we derived a medication list based on the anticholinergic medication classification by Duran and colleagues.^23^ We also included all medications with reported mild or strong anticholinergic/sedative properties and side effects in Dutch Pharmacotherapeutic reference sources in the DBI calculation.^20,21^ Topical preparations, ‘as needed’ medications and medications which lacked a specified dosing regimen in the electronic dispensing records were excluded from the DBI calculation. As the DBI per medication ranges between 0 and 1, depending on the daily dose, our chosen DBI threshold suggests that the patient is prescribed at least 3–4 anticholinergic/sedative medications. In a previous study we found a frail older patient population using about 3–4 anticholinergic/sedative medications being at risk of medication related harm and in need of medication optimization.^9^

### IT-based pharmacist-led intervention

The intervention consisted of five steps. First, the pharmacist obtained a list of eligible patients as described above. For each patient identified with the algorithm, age, sex, DBI, medication profile and medication history were displayed to the pharmacist. Medications that contributed to the patients’ DBI, as well as newly initiated medications along with their date of prescription were highlighted. Second, from the list of displayed patients, pharmacists selected 5–10 patients whom they wished to include in this study. Third, the pharmacists evaluated the medication use, both newly initiated and existing medications, and drafted recommendations to reduce the anticholinergic/sedative load for each of the selected patients. For this evaluation we provided pharmacists an evidence-based guidance document outlining information on rational prescribing for those anticholinergic/sedative medications that are known to be potentially inappropriate in older people, including all newly initiated medications we screened for. Information in the document was based on recent Dutch guidelines and also included recommendations on nonpharmaceutical options.^17^ Fourth, pharmacists discussed recommendations with the GP and if needed, medical specialists. Pharmacists could choose their preferred communication method with the GP, but we advised a face-to-face meeting. Pharmacist and GP agreed who would discuss recommendations for medication changes with the patient, which would be the last step of the intervention.

### Data collection

Data were collected by various methods. Data on the pharmacists, participating pharmacies, patients identified with the algorithm, patients selected for medication review, time taken for each step in the process and the medication review changes proposed were collected *via* an online questionnaire completed by the participating pharmacists. We checked for consistency and completeness of data reported by the pharmacists and based on this we excluded four pharmacists from the analysis.

For each selected patient, the pharmacists reported age, sex, reasons for selection and details of recommendations proposed to the GP. The latter were reported per medication and included type of recommendation (stop/substitute/start medication or change dose, checking indication for use, monitoring lab values or giving other advice), type of prescriber (GP or medical specialist), communication method with GP to discuss recommendations (face-to-face, phone, fax/email or none), whether agreement on the recommendation was reached and if so, who would communicate the recommendations to the patient. If pharmacists had no recommendations for selected patients, they were asked to provide the reasons for this.

The online questionnaire also included structured questions regarding the acceptability of the intervention. Structured questions on a 3-point Likert-scale were used to assess if pharmacists were satisfied with the intervention, if they found it meaningful, if it was considered practical, clear and educational. In addition, the pharmacists were asked if they would like to continue using the intervention in the future following completion of the study.

Data on all medications dispensed between June 2017 and December 2017 for patients who were selected by the pharmacists were provided by SFK. The pharmacists authorized SFK to provide these data. For each patient the medications used on the dispensing date of the newly initiated medication were identified from the dataset and used for the analysis.

We aimed at conducting a structured telephone interview with 1–2 patients per pharmacy to explore the patients’ perspective on reducing their medication use. Each pharmacist asked his/her patients included in this study whether they were willing to participate in a telephone interview. Patients who gave verbal consent to the pharmacist received information about the telephone interview and an informed consent form. Only patients who signed an written informed consent form were interviewed. Each patient interview lasted about 10 min.

### Feasibility

We assessed the number of potentially eligible patients identified with the IT-based tool per pharmacy and the number of falsely identified patients. False identification occurred if the calculated DBI by the module was ⩾2, while in fact the real DBI was <2. This happened due to two problems. First, we detected an error in the online report module, which appeared if the pharmacist ran another algorithm within the online report module. The SFK solved the error within the first month of data collection, but until this time for these pharmacies the online report module did not only include currently used chronic medication in the DBI calculation, but also some anticholinergic/sedative medications that were already stopped. Second, the dispensing data on which the DBI was calculated could include pseudo double medication records. These were records of the same ATC code (level 5), strength and daily dose as another record within one patient with overlapping treatment dates. Pseudo double medication records were a result of early medication dispenses, for example, a patient had not yet finished a medication package, but a new package was already dispensed. We reduced all pseudo double medication records to single medication records. The DBI was recalculated by hand after adjustment of the medication data and compared with the DBI calculated by the module. All demographic characteristics and description of medication use were based on the adjusted dataset.

### Acceptability

Pharmacist and patient acceptability of the intervention was assessed. Pharmacists’ acceptability was assessed by asking the pharmacists whether they found the IT-based intervention meaningful, practical, clear, and educational. We also assessed their willingness to use the intervention in the future. Pharmacists were also asked to report the mean time needed per intervention step per patient.

For the patient perspective on reducing medication use, we determined the number of patients interviewed who expressed a desire to reduce their medication use, who were willing to reduce medication use if the GP would advise this and who were not willing to reduce their medication use even if the GP would advise this.

### Potential effectiveness

Potential effectiveness was assessed in two ways. First, the number of recommendations proposed by the pharmacist and rate of agreement of GP with proposed recommendations was determined. We categorized recommendations into medication changes (stopping, substituting and starting a medication or dosage change) and medication monitoring (checking indication for use, monitoring lab values or giving other advice). We also categorized recommendations for all medications, newly initiated and existing medications. Number of recommendations and rate of agreement was assessed per patient and type of recommendation. Number of patients without recommendations and reasons why were counted and the number of recommendations per ATC level 2 and type of communication to the patient was assessed. Agreement was only counted as such if there was a discussion between pharmacist and GP or specialist. If the prescriber did not respond to the pharmacist’s recommendation, for example, if recommendation was sent *via* email or fax, this was counted as no agreement.

Secondly, we assessed whether patient characteristics, type of medication, type of communication between pharmacist and GP, type of initiating prescriber and level of pharmacotherapy audit meeting with GPs were associated with agreement of the GP with pharmacists’ recommendations. Pharmacotherapy audit meetings were nationally classified into four categories: no structured meetings (level 1), regular meetings without concrete agreements (level 2), regular meetings with concrete agreements (level 3) and regular meetings with evaluating concrete agreements (level 4).^24^ Agreements focused on the prescribing and dispensing of medications.

### Statistical analysis

Descriptive statistics of all data were derived with IBM SPSS Statistics version 25. Factors associated with agreement were analyzed with logistic mixed effects models in MLwiN version 3. Random effects on the level of pharmacy and patient were applied. A univariate analysis on all variables was applied first. Variables with a univariate *p*-value < 0.1 were included in the multivariate analysis. A *p*-value <0.05 was considered significant.

## Results

### Study population

In total, 47 pharmacists from 47 community pharmacies were included in the study. Overall, 305 patients were selected with a median of 6 patients (range 3–10) per pharmacy selected for medication review. The demographic characteristics of pharmacists, pharmacies and patients are shown in Table 1.

**Table 1.** Demographic characteristics pharmacists and patients.

Characteristic	Outcome
**Pharmacists**	***n* = 47**
Age, mean (±SD)	28.3 (2.3)
Sex (% female)	68.1
Working experience, mean years (±SD)	2.0 (1.0)
Working hours per week, median hours (range)	40.0 (24–45)
Pharmacies	*n* = 47
Pharmacists FTE, mean (±SD)	2.3 (1.0)
Number of patients per pharmacy, ***n*** pharmacies per category (%)	
<8000	3 (6.4)
8000–10,000	11 (23.4)
10,000–12,000	13 (27.7)
12,000–14,000	11 (23.4)
>14,000	9 (19.1)
Percentage of patients aged 65+ per pharmacy, ***n*** pharmacies per category (%)	
<20%	11 (23.4)
20–50%	24 (51.1)
>50%	10 (21.3)
Unknown	2 (4.3)
Number of collaborating GPs per pharmacy, mean (±SD)	12.1 (6.3)
Level of pharmacotherapy audit meetings with GPs, ***n*** pharmacies per category (%)	
Level 1: no structured meetings	0 (0.0)
Level 2: regular meetings without concrete agreements	2 (4.3)
Level 3: regular meetings with concrete agreements	30 (63.8)
Level 4: regular meetings with evaluating concrete agreements	13 (27.7)
None	2 (4.3)
**Patients**	***n* = 305**
Age, mean (±SD)	76.5 (8.0)
Sex (% female)	64.0
DBI value at identification, mean (±SD)	3.6 (1.3)
Number of anticholinergic/sedative medications used, mean (±SD)	5.8 (2.1)
**Patients**	***n* = 305**
Number of medications used, mean (±SD)	9.2 (3.3)
Reasons for patient selection for medication review, ***n*** per category (%)*	
Newly initiated medication	142 (46.6)
Risk factors (high age, high DBI, risk medication)	159 (52.1)
Good collaboration with GP	98 (32.1)
Other/no specific reason	88 (28.9)
Top 5 newly initiated anticholinergic/sedative medications, ***n*** patients (%)	
Oxycodone	51 (16.7)
Codeine	43 (14.1)
Tramadol	38 (12.5)
Temazepam	26 (8.5)
Amitriptyline	25 (8.2)
Top 5 used medications per ATC level 1, ***n*** patients (%)	
Cardiovascular system	285 (93.4)
Alimentary tract and metabolism	274 (90.0)
Nervous system	260 (85.2)
Blood and blood-forming organs	164 (53.8)
Respiratory system	83 (27.5)

* Multiple reasons per patient could be selected. A total of 172 patients were selected for one reason, 95 for two reasons, 38 for >2 reasons.

ATC, Anatomical Therapeutical Chemical; DBI, drug burden index; FTE, fulltime equivalent; GP, general practitioner; SD, standard deviation.

### Feasibility

On average, 17.0 [standard deviation (SD) 8.8.0, range 3–32] patients per pharmacy were identified with the IT-based tool. With the calculation of the DBI by hand, we found that 13 selected patients (4.3%) had a real DBI <2. These patients were included due to the error we found in the online report module (*n* = 11) and pseudo double medication records (*n* = 2). In addition, we detected pseudo double medication records for 85 patients (27.9%), but these patients had a DBI ⩾2 even after removing the double medication records. Without adjusting these pseudo double medication records to single records, the mean DBI of patients selected in this study would have been 4.2 (SD 2.0) *versus* 3.6 (SD 1.3) after adjustment.

### Acceptability

A large majority of pharmacists (*n* = 43, 91.5%) were satisfied with the intervention (17 completely, 26 partly), 41 pharmacists (87.2%) found it meaningful (19 completely, 22 partly), 41 pharmacists (87.2%) found it practical (16 completely, 25 partly), 46 pharmacists (97.9%) found it clear (34 completely, 12 partly) and 44 pharmacists (93.6%) found it educational (30 completely, 14 partly). Almost three-quarters of pharmacists (*n* = 33, 70.2%) wanted to keep using the intervention in the future.

The median time investment per patient was 33 min (range 6.5–210). Most time was needed for medication evaluation and drafting of recommendations (median 15 min, range 8–120), then for discussion of recommendations with the GP (median 10, range 5–60), for patient selection a median of 5 min was needed (range 2–60) and the least time was needed to identify patients with the IT-based tool (median 2 min, range 1–25).

Telephone interviews were conducted with 35 patients (10.7%). One in five patients (*n* = 8, 22.9%) reported that they wished to stop one or more medications or would stop on GP’s advice (*n* = 22, 62.9%). There were five patients (14.3%) who did not want to stop any medication, even if advised by the GP.

### Potential effectiveness

Recommendations were proposed for 212 patients (69.5%), a mean of 1.7 (SD 0.9) per patient. Recommendations included medication changes (169 patients), medication monitoring (24 patients) or both (19 patients). Overall, the GP agreed with pharmacists’ recommendations in 108 patients (35.4%) and with recommendations to change medications in 97 patients (31.8%).

Most recommendations were proposed for opioids (ATC N02A, 16.8%), such as oxycodone and tramadol (respectively 40.7% and 42.4%), antidepressants (ATC N06A, 13.1%), such as amitriptyline (52.2%), anxiolytics (ATC N05B, 10.3%), such as oxazepam (58.3%), and sedatives (ATC N05C, 9.7%), such as temazepam (67.6%). A detailed overview of all recommendations proposed on medication grouped by ATC level 2 can be found in additional file 1.

For 93 patients (30.5%) no recommendations were proposed. Reasons for not proposing an intervention were that no medication optimization was possible, for example, the medication being for short term use or patient was already on tapering scheme (62 patients), the pharmacist knew beforehand that either patient or GP would not accept any medication recommendation (15 patients), medication recommendations were difficult as medication was of a specialist nature (9 patients) or due to other reasons, for example, patient had died (6 patients). For one patient the pharmacist did not report the reason for not proposing a recommendation.

In total 351 recommendations were proposed, of which 148 (48.5%) were agreed with by the GP. For 13 of 351 recommendations (4.3%) the medical specialist was contacted. Stopping a medication or substitution by a safer alternative were the most commonly proposed recommendations, respectively 41.3 and 32.5% of the total recommendations. The rate of agreement for stopping or substituting a medication was higher for newly initiated medications (57.8% and 35.3% agreement) than for existing medications (30.9% and 24.1% agreement). Agreement to stop a medication was reached in 17.7% of recommendations (62 of 351), in 19.8% of recommendations for newly initiated medications (37 of 187) and in 15.2% of recommendations for existing medications (25 of 164), Table 2.

**Table 2.** Type of recommendations by pharmacist and rate of agreement by general practitioner.*

Recommendation	Total (***n*** = 351)		Newly initiated medications (***n*** = 187)		Existing medications (***n*** = 164)	
	Proposed *n* (% of total proposed)	Agreed *n* (% of proposed)	Proposed *n* (% of total)	Agreed *n* (% of proposed)	Proposed *n* (% of total)	Agreed *n* (% of proposed)
Medication changes						
Stop	145 (41.3)	62 (42.8)	64 (34.2)	37 (57.8)	81 (49.4)	25 (30.9)
Substitute	114 (32.5)	37 (32.5)	85 (45.5)	30 (35.3)	29 (17.7)	7 (24.1)
Dose adjustment	32 (9.1)	15 (46.9)	14 (7.5)	5 (35.7)	18 (11.0)	10 (55.6)
Start	9 (2.6)	5 (55.6)	0 (0)	–	9 (5.5)	5 (55.6)
Subtotal	300 (85.5)	119 (39.7)	163 (87.2)	72 (44.2)	137 (83.5)	47 (34.3)
Medication monitoring						
Check lab values	13 (3.7)	12 (92.3)	0 (0)	–	13 (7.9)	12 (92.3)
Additional information on medication use (e.g. advice or check indication)	38 (10.8)	17 (44.7)	24 (12.8)	11 (45.8)	14 (8.5)	6 (42.9)
Subtotal	51 (14.5)	29 (56.9)	24 (12.8)	11 (45.8)	27 (16.5)	18 (66.7)
Total	351 (100)	148 (42.2)	187 (100)	83 (44.4)	164 (100)	65 (39.6)

* for 13 of 351 recommendations (4.3%) a medical specialist was contacted.

Of the 148 recommendations with agreement, discussion with the patient was done by the GP (*n* = 54, 36.5%), pharmacist (*n* = 46, 31.1%) or someone else (*n* = 9, 6.1%). In some cases, there was no communication with the patient as he/she was not reachable by phone (*n* = 7, 4.7%) or no discussion was needed (for example, lab-value check; *n* = 15, 10.1%). For 17 recommendations (11.5%) the pharmacists did not report who contacted the patient.

GP agreement with proposed recommendations was more likely for recommendations on cough and cold preparations (codeine; odds ratio (OR) 3.30; 95% confidence interval (CI) 1.14–9.57) or medication initiated by a medical specialist (OR 2.85; 95% CI 1.19–6.84). Furthermore, a less established working collaboration between pharmacist and GP’s resulted in less agreement with recommendations compared with well-established collaboration (OR 0.15; 95% CI 0.02–0.97), Table 3.

**Table 3.** Factors associated with GP agreement with recommended medication changes.

Factor	Univariate		Multivariate	
	OR (95% CI)	*p*-value	OR (95% CI)	*p*-value
Patient characteristics				
Age	0.98 (0.95–1.01)	0.264	NA	NA
Sex	0.67 (0.40–1.14)	0.142	NA	NA
DBI	0.99 (0.82–1.19)	0.885	NA	NA
Number of medications	1.01 (0.94–1.09)	0.844	NA	NA
Type of medication				
Newly initiated medication	1.75 (1.02–2.99)	0.042*	1.47 (0.84–2.58)	0.182
Drugs for acid related disorders (ATC code A02)	1.07 (0.39–2.97)	0.898	NA	NA
Urologicals (ATC code G04)	0.86 (0.28–2.57)	0.794	NA	NA
Analgesics (ATC code N02)	1.60 (0.85–3.00)	0.142	NA	NA
Psycholeptic (ATC code N05)	0.52 (0.29–0.93)	0.027*	0.58 (0.32–1.07)	0.082
Psychoanaleptic (ATC code N06)	0.55 (0.26–1.17)	0.119	NA	NA
Cough and cold preparations (ATC code R05)	4.71 (1.64–13.50)	0.004*	3.30 (1.14–9.59)	0.028*
Type of communication between pharmacist and GP				
Face-to-face	2.10 (0.58–7.60)	0.262	NA	NA
Telephone	1.41 (0.39–5.05)	0.613	NA	NA
Fax/email	1.43 (0.37–5.55)	0.617	NA	NA
None	Ref	Ref	NA	NA
Initiating prescriber				
Medical specialist	2.44 (1.03–5.79)	0.042*	2.85 (1.19–6.84)	0.019*
GP	Ref	Ref	Ref	Ref
Pharmacotherapy audit meeting pharmacist/GPs				
None to level 2^a^	0.13 (0.02–0.82)	0.030*	0.15 (0.02–0.97)	0.047*
Level 3^b^	1.10 (0.55–2.19)	0.809	NA	NA
Level 4^c^	Ref	Ref	Ref	Ref

* Statistically significant.

a no (structured) meetings (level 1) or regular meetings without concrete agreements (level 2).

b regular meetings with concrete agreements (level 3).

c regular meetings with concrete agreements and evaluation (level 4).

Variables with a univariate *p*-value <0.1 were included in the multivariate analysis.

ATC, Anatomical Therapeutical Chemical; CI, confidence interval; DBI, drug burden index; GP, general practitioner; NA, not applicable, not included in the multivariate analysis; OR, odds ratio; Ref, reference.

## Discussion

### Key findings

The innovative IT-based pharmacist-led intervention targeting newly initiated anticholinergic/sedative medications was feasible, acceptable and potentially effective. Pharmacists were able to identify a considerable number of older patients in need of medication optimization with the IT-based tool. Acceptability of the intervention was high both among pharmacists and patients. The potential effectiveness of the intervention appears high with one or more recommendations being proposed for over two-thirds of patients and agreement of GP with pharmacists’ recommendations for one-third of all patients. Agreement was more likely for recommendations on codeine use, for medications initiated by a medical specialist, and when pharmacist and GP had a well-established working collaboration.

### Comparison with other studies

The fragile older population with a high anticholinergic/sedative load included in this study was comparable with the population selected in our previous randomized controlled trial on pharmacist-led medication review in terms of age, sex, DBI and medication use.^9^ While the previous study found that pharmacist-led medication review was not effective in reducing anticholinergic/sedative load associated with chronic medication, our new approach targeting newly initiated anticholinergic/sedative medications appears more successful, especially for newly initiated medications including anxiolytics, hypnotics and antidepressants. While our approach is innovative in the pharmacy setting, our results are comparable with a study in the general practice setting, which found that newly initiated benzodiazepines and tricyclic antidepressants, were more likely to be successfully reduced by GPs than long-term used hypnotics.^25^ GP agreement with pharmacists’ recommendations in our study was comparable with others. In line with these studies, agreement seemed higher when GP and pharmacist had a well-established working collaboration.^26^

The GP was more likely to agree with recommendations for medications with unknown or questionable efficacy and a high side effect profile, such as codeine.^27^ We found that agreement was higher for medications initiated by a medical specialist compared with GP. This was surprising as previous literature found that medical specialists in general are less likely to agree with pharmacist recommendations compared with primary care physicians.^28^ However, most recommendations in our study focused on psychotropic medication and contacting a medical specialist for these medications might be preferable.

### Strengths and limitations

We developed and evaluated an innovative intervention performed in a relatively large homogenous group of motivated, early-career pharmacists who had access to the full medication records for their patients and who were trained in pharmaceutical patient care. The evaluation conducted was robust, following accepted guidance for the development and evaluation of complex health care interventions and included feasibility, acceptability and potential effectiveness in a large number of pharmacists and patients.^12^ This evaluation provides valuable information for further development and testing of the intervention. The intervention was designed for the convenience of the pharmacist and pharmacists could adapt it to fit his or her practice in the real-world setting. Analyzing the impact of the intervention, we identified the number of recommendations and classified those in a meaningful way, distinguishing between medication changes and medication monitoring.

Some limitations need to be taken into consideration when interpreting our results. First, due to the nature of our study it was not possible to perform a follow-up meeting. We therefore do not know whether all planned medication changes were implemented. We report on the agreement of the GP with pharmacists’ recommendations, which may overestimate actual implemented medication changes. Also, it was outside the scope of our study to explore to what extent pharmacist or GP communicated recommendations with the patient. Second, we do not know whether all steps of the intervention were followed in the proposed order, for example, some patients could have been contacted before discussion with the GP. However, this was a result of the real-world nature of the intervention and allowing some flexibility in the order of steps is likely to make a strategy more pragmatic in clinical practice. Third, using dispensed medication records has disadvantages, such as pseudo double medication records which resulted in a small number of false positive identification of patients with pseudo double medication records with the same ATC level 5 medications. There could have also been patients with pseudo double medication records with different ATC codes, for example, patients who switched to another medication with similar effects. Fourth, wide CIs suggest that our data sample was not large enough to draw strong conclusions about the factors associated with GP agreement with recommended medication changes. But we believe that our findings are a basis for further refinement of the intervention. Finally, this project was part of the pharmacists’ post-graduate training and all pharmacists should have been able to perform the intervention. However, we had to exclude four pharmacists as data provided from these pharmacies was inconsistent. We think that this is a reflection of real-world practice, in which practicalities, for example, building renovations, changing IT system, sickness, holidays, lack of personnel, but perhaps also lack of motivation may affect the performance of interventions. Furthermore, there might be a difference in motivation of using the IT-based tool between the young pharmacists in our study compared with more experienced pharmacists in practice.

### Conclusions and implications for practice and further research

The pharmacist-led IT-based intervention as performed in this study, appears feasible, acceptable and potentially effective. Pharmacists needed on average about half an hour to perform the intervention and in one out of three patients the GP agreed with pharmacists’ recommendations to change medication. Therefore, when extrapolating, about 1.5 h was needed to prevent an increase in anticholinergic/sedative load in one patient. Our results suggest some refinements of the intervention should be considered prior to upscaling. Our study used the algorithm retrospectively to identify patients over the past month who could be considered for a medication review. In line with the current use of IT-based drug therapy alerts in Dutch pharmacy practice, the algorithm should be fully integrated in the pharmacy information system. This way it will operate prospectively with the system deploying an alert for a newly initiated anticholinergic/sedative medication that would increase the patient’s total anticholinergic/sedative load above the specific threshold at the time the prescription is presented for initial supply. Further therapeutic advice for reducing the load should be directly displayed alongside the alert. This way, the pharmacist is able to propose and discuss recommendations with the GP prior to dispensing the medication. These refinements will likely increase the rate of implementation of recommendations, as the medication change is being implemented before the patient has commenced treatment with the newly prescribed medication. Secondly, as no consensus-based list of anticholinergic/sedative medication is available,^29^ we included a broad range of medications with mild and strong anticholinergic/sedative properties or reported side effects. Most recommendations in our study were proposed for medications with strong anticholinergic/sedative properties, such as psychotropic and bladder antimuscarinics, only a few recommendations were proposed for medications with mild or unknown anticholinergic/sedative properties, like cardiovascular medication. We suggest a refinement of our list, including only medications with known anticholinergic/sedative properties and frequently reported anticholinergic/sedative side effects, this may reduce alert fatigue.^30^ Furthermore, while we used the DBI to calculate the anticholinergic/sedative load, other tools have been developed, among those, one that shows promising results.^31,32^ Finally while the feasibility, acceptability and potential effectiveness of the intervention appears high, the cost-effectiveness and implementation of medication recommendations and long-term medication changes in combination with relevant patient outcomes, like geriatric outcomes (e.g. fall risk, frailty and cognitive function) and adverse events (e.g. drug-related hospital admission)^33^ should be evaluated in a real-world randomized controlled trial in community pharmacies preferably with high level collaboration with GPs.

## Supplemental Material

Additional_file_Therapeutic_advances_in_drug_safety_(1) – Supplemental material for Feasibility, acceptability and potential effectiveness of an information technology-based, pharmacist-led intervention to prevent an increase in anticholinergic and sedative load among older community-dwelling individuals Click here for additional data file. Supplemental material, Additional_file_Therapeutic_advances_in_drug_safety_(1) for Feasibility, acceptability and potential effectiveness of an information technology-based, pharmacist-led intervention to prevent an increase in anticholinergic and sedative load among older community-dwelling individuals by Helene G. van der Meer, Hans Wouters, Martina Teichert, Fabiënne Griens, Jugoslav Pavlovic, Lisa G. Pont and Katja Taxis in Therapeutic Advances in Drug Safety
