# Supplementary material for: Feasibility, acceptability and potential effectiveness of an information technology-based, pharmacist-led intervention to prevent an increase in anticholinergic and sedative load among older community-dwelling individuals
Source: Ther Adv Drug Saf. 2018 Oct 30;10:2042098618805881. doi: 10.1177/2042098618805881 (PMC6463339; doi:10.1177/2042098618805881)
Supplement: Additional_file_Therapeutic_advances_in_drug_safety_(1) – Supplemental material for Feasibility, acceptability and potential effectiveness of an information technology-based, pharmacist-led intervention to prevent an increase in anticholinergic and sedative load among older community-dwelling indivi [file Additional_file_Therapeutic_advances_in_drug_safety_(1).pdf]

Additional file 1: Type of recommendations proposed by pharmacists on medication grouped by ATC level 2 and rate of agreement by general practitioner

All medications			Newly initiated medications			Existing medications		
ATC code	Proposed n (% of total)	Agreed n (% of proposed)	ATC code	Proposed n (% of total)	Agreed n (% of proposed)	ATC code	Proposed n (% of total)	Agreed n (% of proposed)
Medication changes: stop								
N05	45 (12.8)	14 (31.1)	N02	27 (14.4)	17 (63.0)	N05	24 (14.6)	4 (16.7)
N02	29 (8.3)	19 (65.5)	N05	21 (11.2)	10 (47.6)	A02	14 (8.5)	5 (35.7)
G04	15 (4.3)	6 (40.0)	G04	6 (3.2)	3 (50.0)	G04	9 (5.5)	3 (33.3)
A02	14 (4.0)	5 (35.7)	R05	4 (2.1)	3 (75.0)	N06	9 (5.5)	2 (22.2)
N06	13 (3.7)	4 (30.8)	N06	4 (2.1)	2 (50.0)	N07	4 (2.4)	0 (0)
						C03	4 (2.4)	3 (75.0)
Subtotal	145 (41.3)	62 (42.8)	Total	64 (34.2)	37 (57.8)	Total	81 (49.4)	25 (30.9)
Medication changes: substitute								
N05	30 (8.5)	6 (20.0)	N06	20 (10.7)	6 (30.0)	N05	10 (6.1)	3 (30.0)
N06	27 (7.7)	8 (29.6)	N02	21 (11.2)	9 (42.9)	N06	7 (4.3)	2 (28.6)
N02	23 (6.6)	9 (39.1)	N05	20 (10.7)	3 (15.0)	C07	4 (2.4)	0 (0)
R05	15 (4.3)	9 (60.0)	R05	15 (8.0)	9 (60.0)	N02	2 (1.2)	0 (0)
R06	5 (1.4)	2 (40.0)	R06	4 (2.1)	2 (50.0)	C09	3 (1.8)	1 (33.3)
Subtotal	114 (32.5)	37 (32.5)	Total	85 (45.5)	30 (35.3)	Total	29 (17.7)	7 (24.1)
Medication changes: dose adjustment								
N05	10 (2.8)	4 (40.0)	N05	5 (2.7)	2 (40.0)	N05	5 (3.0)	2 (40.0)
N02	6 (1.7)	1 (16.7)	N02	4 (2.1)	0 (0)	A02	5 (3.0)	2 (40.0)
A02	5 (1.4)	2 (40.0)	N06	3 (1.6)	1 (33.3)	N02	2 (1.2)	1 (50.0)
N06	3 (0.9)	1 (33.3)	R05	2 (1.1)	2 (100)	A11	2 (1.2)	2 (100)
R05	2 (0.6)	2 (100.0)				C07	1 (0.6)	1 (100)

A11	2 (0.6)	2 (100.0)								
Subtotal	32 (9.1)	15 (46.9)		Total	14 (7.5)	5 (35.7)		Total	18 (10.9)	10 (55.6)
Medication changes: start										
C10	3 (0.9)	1 (33.3)						C10	3 (1.8)	1 (33.3)
A12	2 (0.6)	2 (100)						A12	2 (1.2)	2 (100)
A06	2 (0.6)	1 (50.0)						A06	2 (1.2)	1 (50.0)
C03	1 (0.3)	0 (0)						C03	1 (0.6)	0 (0)
A02	1 (0.3)	1 (100)						A02	1 (0.6)	1 (100)
Subtotal	9 (2.6)	5 (55.6)		Total	0 (0)	-		Total	9 (5.5)	5 (55.6)
Medication monitoring: check lab-values										
C10	5 (1.4)	5 (100)						C10	5 (3.0)	5 (100)
C03	3 (0.9)	3 (100)						C03	3 (1.8)	3 (100)
B03	2 (0.6)	1 (50.0)						B03	2 (1.2)	1 (50.0)
C09	2 (0.6)	2 (100)						C09	2 (1.2)	2 (100)
C07	1 (0.3)	1 (100)						C07	1 (0.6)	1 (100)
Subtotal	13 (3.7)	12 (92.3)		Total	0 (0)	-		Total	13 (7.9)	12 (92.3)
Medication monitoring: additional information on medication use										
Unknown	19 (5.4)	0 (0)		Unknown	11 (5.9)	0 (0)		Unknown	8 (4.9)	0 (0)
G04	6 (1.7)	5 (83.3)		G04	5 (2.7)	4 (80.0)		A02	2 (1.2)	2 (100)
N05	4 (1.1)	4 (100)		N05	4 (2.1)	4 (100)		C08	2 (1.2)	2 (100)
N06	3 (0.9)	3 (100)		N06	2 (1.1)	2 (100)		G04	1 (0.6)	1 (100)
C08	3 (0.9)	3 (100)		C08	1 (0.5)	1 (100)		N06	1 (0.6)	1 (100)
A02	2 (0.6)	2 (100)		N02	1 (0.5)	0 (0)				
Subtotal	38 (10.8)	17 (44.7)		Total	24 (12.8)	11 (45.8)		Total	14 (8.5)	6 (42.9)
Total recommendations										

N05	89 (25.4)	28 (31.5)		N02	53 (28.3)	26 (49.1)		N05	39 (23.8)	9 (23.1)
N02	59 (16.8)	29 (49.2)		N05	50 (26.7)	19 (38.0)		A02	22 (13.4)	10 (45.5)
N06	46 (13.1)	16 (34.8)		N06	29 (15.5)	11 (37.9)		N06	17 (10.4)	5 (29.4)
G04	23 (6.6)	12 (52.2)		R05	21 (11.2)	14 (66.7)		C10	11 (6.7)	7 (63.6)
A02	22 (6.3)	10 (45.5)		G04	13 (7.0)	8 (61.5)		G04	10 (6.1)	4 (40.0)
R05	21 (6.0)	14 (66.7)						C03	8 (4.9)	6 (75.0)
Total	351 (100)	148 (42.2)		Total	187 (100)	83 (44.4)		Total	164 (100)	65 (39.6)

ATC = Anatomical Therapeutical Chemical.
